# Longitudinally changed diet quality scores and their association with type 2 diabetes mellitus and cardiovascular diseases in the EPIC-Potsdam study

**DOI:** 10.1038/s41598-024-63899-8

**Published:** 2024-06-17

**Authors:** Franziska Jannasch, Daniela V. Nickel, Olga Kuxhaus, Matthias B. Schulze

**Affiliations:** 1 Department of Molecular Epidemiology, German Institute of Human Nutrition Potsdam-Rehbruecke, Arthur-Scheunert-Allee 114-116, 14558 Nuthetal, Germany; 2 NutriAct Competence Cluster for Nutrition Research, Berlin-Potsdam, Germany; 3 German Center for Diabetes Research (DZD), München-Neuherberg, Germany; 4 Institute of Nutritional Science, University of Potsdam, Nuthetal, Germany

**Keywords:** Diet quality scores, Mediterranean diet, Type-2-diabetes mellitus, Cardiovascular disease, Cardiology, Diseases, Endocrine system and metabolic diseases, Nutrition, Risk factors

## Abstract

Association analyses between longitudinal changes in diet quality scores (DQIs) and cardiometabolic risk remain scarce. Hence, we aimed to investigate how changes in two DQIs are associated with incident type 2 diabetes (T2D), myocardial infarction (MI) and stroke in the EPIC-Potsdam study. Changes in the Mediterranean Pyramid Score (MedPyr) and Healthy Diet Score (HDS) over 7 years from baseline (1994–1998) to follow-up 3 (2001–2005) were investigated in 23,548 middle-aged participants. Adjusted Cox Proportional Hazards Regression models were applied to investigate associations between changes in MedPyr and HDS and chronic disease incidence. More than 60% of the participants increased both DQIs more than 5%. Within a median follow-up time of 5 years 568 cases of T2D, 171 of MI, 189 of stroke were verified. An increased compared to stable MedPyr was associated with lower T2D risk (HR 0.74; 95% CI 0.59–0.92), while a decreased MedPyr was associated with higher stroke risk (HR 1.67; 95% CI 1.02–2.72). A decreased compared to stable HDS was associated with higher stroke risk (HR 1.80; 95% CI 1.02–3.20). The findings contribute further evidence on advantages of changing dietary intake towards a Mediterranean Diet. Although baseline HDS adherence was associated with T2D and stroke risk, longitudinal changes in HDS were only significantly associated with stroke risk.

## Introduction

Besides the investigation of individual foods and nutrients, the study of dietary patterns acts as a complementary strategy to disentangle the complexity of human diet. It has the advantage to consider the interrelation of food intake and to represent the cumulative exposure to different diet components, potentially leading to stronger effects on health than any single component^1^. Numerous prospective studies have evaluated associations between dietary patterns and cardiometabolic diseases^2,3^. Regarding a priori (hypothesis-based) dietary patterns (DP), evidence is strongest for the Mediterranean diet^2,4,5^, but also the Alternative Healthy Eating Index (AHEI) and Dietary Approaches to Stop Hypertension (DASH) were associated with a reduced risk of type 2 diabetes (T2D) and cardiovascular diseases (CVD)^2,3,6^. Noteworthy, studies conducted outside the Mediterranean region, thus in study populations which generally follow different dietary habits, e.g. European Prospective Investigation into Cancer and Nutrition (EPIC)-Potsdam study among others, also observed lower cardiometabolic risk for participants following a Mediterranean diet^7–9^. Beneficial effects of the Mediterranean diet are strongly supported by the Prevención con Dieta Mediterránea (PREDIMED) randomized trial, which showed lower CVD risk with this diet, being either supplemented with extra virgin olive oil or with nuts^10^.

Previous studies mostly used dietary information collected from only one time point. However, using dietary information repeatedly collected during follow-up could be used to reduce measurement error due to intra-individual variation^11^. Furthermore, in terms of supporting dietary guidelines, it is of particular interest to investigate, if an actual change in dietary intake is associated with the incidence of chronic diseases. To our knowledge, few studies have already investigated the effect of changes in DPs over time on chronic disease risk: A decrease in diet quality assessed by the AHEI was related to higher T2D risk, while an increase in diet quality was related to lower risk in three US cohorts^12^. Similarly, longitudinal increases in the diet quality scores alternative Mediterranean diet (aMED), AHEI and DASH were associated with a lower CVD risk^13^. In European cohorts, most investigations on longitudinal changes of DP were restricted to associations with intermediate cardiometabolic risk factors^14–16^ and one study used diet data from repeated assessments, but calculated the cumulative average and its association with cardiovascular disease risk^9^.

To close the knowledge gap on a potential association of changes in dietary behavior with chronic disease endpoints in European cohorts, we aimed to investigate the longitudinal change in diet quality scores and its association with incident T2D, MI and stroke in the EPIC-Potsdam study, a prospective cohort with repeated dietary assessments and follow-up on cardiometabolic diseases.

## Subjects and methods

### Study population

Participants were recruited between 1994 and 1998 from the general population living in Potsdam and surrounding areas, with 16,644 women aged 35 to 65 years and 10,904 men aged 40 to 65 years participating^17^. At baseline, participants were examined by trained personnel for anthropometric assessment and answered questions on their prevalent diseases, diet and lifestyle via interviewer-based and self-administered questionnaires^18^. Informed consent was obtained from all participants and the study was approved by the Ethical Committee of the State of Brandenburg, Germany and follows the recommendations of the Declaration of Helsinki. During follow-up, the participants were contacted again every 2–3 years to identify incident cases of the respective diseases of interest^19^, and were asked to fill in a questionnaire on their habitual dietary intake at the third follow-up (FUP3) between 2001 and 2005^20^. For this analysis, participants with information on their vital status until the fifth follow-up (FUP5) (2007–2009) were included. The follow-up rates ranged between 90 and 96% for each follow-up round. At FUP3 26,345 participants were still active (Supplementary Fig. 1). From these, we excluded participants with incomplete questionnaire data (n = 2147). Further exclusions on missing diet (n = 10) and covariates (n = 640) were done, resulting in a study population of n = 23,548 participants. We were interested in the change in diet from baseline to FUP3, and the subsequent occurrence of incident T2D and CVD until FUP5. Hence, we excluded participants who reported to be diagnosed with T2D, MI or stroke at baseline and those who developed T2D (n = 2155), MI (n = 597) or stroke (n = 461) until FUP3 for the disease-specific association analyses (Supplementary Fig. 1).

### Dietary assessment

The habitual intake of diet was assessed with a semi-quantitative FFQ at baseline (FFQ_0_) comprising 149 food items. The frequency of intake was inquired with 10 categories ranging from “I don’t eat it” to “5 times per day or more”. Additional pictures of portion sizes supported participants to estimate their consumed quantity. The respective standard portion size was adopted from a list of food items, which was developed in the framework of the MONItoring of trends and determinants of CArdiovascular disease (MONICA) project in Augsburg, Germany^21^. With regard to selected food groups prone to misreporting due to e.g. seasonal variance, summation questions on those food groups were used to determine a correction factor for the calculation of the overall habitual intake in g/day. At FUP3, participants were asked again to complete an updated FFQ (FFQ_1_), which comprised a shortened list of 105 food items. The update of the FFQ was conducted systematically to restrict the number of inquired food items to those explaining maximum variance in food groups and selected nutrients, and to include new food items to consider trends in food intake over time^20^. Portion sizes were provided by common household measures and the frequency of consumption was asked in 5 to 10 response categories, depending on the inquired food items.

Systematic differences between the two FFQs were investigated and corrected. Details are given in the Supplementary Methods “Systematic investigation of differences between FFQ_0_ and FFQ_1_” and Supplementary Table 1.

### Choice of diet quality scores and calculation of their longitudinal changes

In our previous investigation of the association between baseline diet quality scores with the incidence of T2D, myocardial infarction (MI) and stroke in the EPIC-Potsdam study, we focused on the Mediterranean diet, using a scoring originally derived by Trichopoulou et al. (tMDS)^22^ and the Mediterranean Pyramid (MedPyr) score, and the Nordic diet^7^. In the present study, investigating longitudinal changes in adherence to diet quality scores, we considered only the MedPyr to reflect a Mediterranean diet, as it was more clearly associated with lower incident T2D in EPIC-Potsdam in the former analysis^7^. Instead of the Nordic diet, which was not associated with the chronic diseases in our study population^7^, we decided to investigate a newly developed diet quality score for healthy eating (Healthy Diet Score—HDS)^23^. This considers the current German dietary guidelines and latest evidence of beneficial and detrimental associations of food groups with the chronic diseases of interest. The diet scores have been investigated according to their relative validity and reliability in a validation study (n = 134) embedded in EPIC-Potsdam^23,24^. For both scores, reliability and relative validity were moderate. The MedPyr showed a mean difference of 0.03 score points and a correlation of r = 0.62 based on two similar FFQs applied one year apart, while the HDS showed a mean difference of 0.03 score points and a correlation of r = 0.53. Relative validity was lower but still moderate: the FFQ derived data were compared to the mean of 12 24-h dietary recalls within one year (MedPyr: mean difference − 0.31 and r = 0.45; HDS: mean difference 0.29 and r = 0.43).

Details of the construction of both scores were described in previous publications. Briefly, the MedPyr comprises 15 food groups reflecting guidelines of the Mediterranean pyramid^7,9^ (Supplementary Table 2), while the HDS comprises 10 food groups, which were selected based on German dietary guidelines complemented by latest evidence of their beneficial and detrimental potential for the development of chronic diseases^23^ (Supplementary Table 3). While the MedPyr theoretically ranges from 0 to 15 points, the HDS ranges from 0 to 10 points.

The relative change in diet quality scores between baseline and FUP3 (in percent) was calculated as the absolute difference between the scores from FUP3 and baseline, divided by the baseline score and multiplied by 100 (Formula 1).

1 $${diet change}_{perc}=\frac{{diet quality score}_{FUP3}-{diet quality score}_{Baseline}}{{diet quality score}_{Baseline}}*100$$

A change of 5% or less in the two diet quality scores was categorized as “stable”, while more than 5% decrease in the diet quality scores was categorized as “decrease” and more than 5% increase in the scores as “increase”, partly comparable to the approaches in previous U.S. based cohorts^12,25^.

An overview of the food groups at baseline and at FUP3 across the change categories of both diet quality indices was provided in Supplementary Tables 4, 5.

### Covariate assessment

Baseline and FUP3 information on socio-demographic covariates like age, sex and occupation, lifestyle factors like alcohol consumption and smoking behavior, as well as self-reported hypertension were obtained by self-administered questionnaires and an interview (at baseline), except for physical activity (lastly updated information at FUP2) and education (only baseline information). Categories of highest educational level were “currently in training/no certificate or skill”, “professional school (vocational training)”, and “college or higher education”. Occupational status was a variable with the three categories “employed”, “unemployed” and “retired”. Smoking status was categorized as “never smoker”, “former smoker” and “current smoker”. If the updated information on smoking behavior was not reported at FUP3, the missing information was imputed based on the reported information at a later follow-up. Physical activity was obtained as combined information of leisure time sports activities and biking, both in hours/week. Lifetime alcohol consumption combined the information on alcohol intake during life course (at the age 20, 30 and 40) and the intake estimate from baseline and FUP3, respectively^26^.

The anthropometrics of the participants were assessed by trained staff at baseline, who followed a standard protocol with a strict quality control^27^. Body weight was measured by electronic digital scales, accurate to 0.1 kg and heights were measured to the nearest 0.1 cm using a flexible anthropometer^28^. At FUP3 body weight was self-reported by the participants. The Body-Mass-Index (BMI) was calculated by dividing the body weight in kg by the square of height in m.

### Outcome ascertainment

Incident T2D, MI and stroke were ascertained by self-reports of the disease, disease-relevant medication or diagnosis as a reason for change in diet at the follow-ups. Further potential cases were obtained from medical databases, death certificates or other sources like physicians or clinics. All potential cases had to be verified by the primary care physician, including the correct diagnosis, the diagnostic test and exact date of diagnosis^19^. The respective International Classification of Diseases (ICD-10) codes I21 for MI, I60, I61, I63 and I64 for stroke and E11 for T2D were used.

### Statistical analyses

Characteristics of the participants are described across the diet quality score change categories as mean ± standard deviation, if normally distributed and as median (interquartile range), if the assumption of normal distribution was not fulfilled. Categorical variables are presented as relative proportions.

Since the HDS has not been investigated according to its association with chronic disease risk before, an analysis was conducted, where the association of baseline adherence to the HDS (in quintiles) as exposure with the incidence of all three outcomes until the FUP5 (mean follow-up time: 11 years) was investigated. For this, a Cox Proportional Hazards Regression model was set up, where the time between age at recruitment and age at exit was used as underlying time scale and the model was stratified by integers of age. To account for potential covariates, the model was adjusted for sex, education, occupational status, vitamin supplementation, self-reported hypertension, smoking status, physical activity, lifetime alcohol consumption, total energy intake and BMI, all covariates respectively assessed at baseline to achieve comparability to a previous investigation on the association between adherence to other diet quality scores and cardiometabolic diseases in our study population^7^.

Associations between the change of both respective diet quality scores as exposure and the incident chronic diseases as outcome were calculated as multivariable-adjusted hazard ratios (HR) with 95% confidence intervals by Cox Proportional Hazards Regression models. The change in diet quality scores was modeled as (a) continuous exposure per 20%-increase (corresponds to 3-points increase for MedPyr; 2 points increase for HDS) and (b) the percentual change as categorical exposure, setting “stable” as reference category to account for potential trends in decreases or increases. The dependent time variable was the time between age at FUP3 and age at exit (either date of diagnosis, date of death or censoring at the end of FUP5). The models were stratified by integers of age to be less sensitive to violations of the HR by different age-dependent risk at analysis baseline. Model 1 was adjusted for sex, the respective diet quality score and education at baseline, while baseline and updated FUP3 information was used for occupational status, self-report of hypertension, smoking status and lifetime alcohol consumption, total energy intake, and physical activity (taken from FUP2, where the information was available), respectively. Model 2 was additionally adjusted for baseline BMI and BMI at FUP3 to account for potential confounding on the diet change-disease-association. Schönfeld residuals were calculated to check the proportional hazards assumptions. Non-linear associations between the change in diet quality scores and the outcomes of interest were evaluated with restricted cubic splines (RCS) and a Wald χ^2^ test.

To assess potential effect measure modification of the association between the change in diet quality and the respective outcomes by sex^29,30^ or by baseline diet quality score, stratified analyses were conducted. Furthermore, multiplicative interaction terms with the respective diet quality scores were added to Model 2 and tested in a Wald test for significant interaction. To account for potential misreporting, participants with implausible energy intake (women: < 500 kcal/day, > 3500 kcal/day; men: < 800 kcal/day, > 4200 kcal/day) at baseline (n = 410) and at FUP3 (n = 265) were excluded in a sensitivity analysis. Those participants, who reported a change in diet at FUP3 due to weight gain (n = 5536), high blood pressure (n = 143), dyslipidemia (n = 596) or diabetes (n = 766) within the previous 2 years, were excluded in further sensitivity analyses. To investigate, if potential beneficial effects of the MedPyr score changes on the outcomes were driven by moderate alcohol consumption, this component was excluded in a sensitivity analysis. All analyses were performed with SAS Software version 9.4 (SAS Institute Cary, NC).

### Ethics approval

This study was performed in line with the principles of the Declaration of Helsinki. All study procedures were approved by the Ethical Committee of the State of Brandenburg.

### Consent to participate

Participants agreed to participate in the EPIC-Potsdam study by signing the informed consent form.

## Results

Table 1 shows that more than 60% of the 23,548 included participants increased adherence to both diet quality scores by more than 5% between baseline and FUP3 (median time span: 6.6 years), while approximately similar proportions of participants decreased their diet quality scores or remained relatively stable. Participants in the increase-category for both diet quality scores were less likely to be men, started with a lower diet quality score at baseline and lower total energy intake. No differences across the change categories were observed for baseline age, BMI or physical activity. While alcohol consumption was considerably lower in participants, who increased adherence to MedPyr, the consumption was not different between participants, who increased adherence to HDS compared to those who remained stable. The increase in both diet quality scores was in line with the higher rate of self-reported change in diet in the previous 2 years at FUP3.

**Table 1:** Characteristics of the EPIC-Potsdam study population across the categories of diet quality score changes (n = 23,548).

	Change in Mediterranean Pyramid			Change in Healthy Diet Score		
	Decrease (> 5%)	Stable (± ≤ 5%)	Increase (> 5%)	Decrease (> 5%)	Stable (± ≤ 5%)	Increase (> 5%)
n	4104	4291	14,854	3332	3425	16,492
Male, %	49.7	41.6	34.8	46.4	34.8	38.0
Age at baseline, years	50.4 ± 8.60	50.1 ± 8.81	50.6 ± 9.09	49.7 ± 8.93	50.4 ± 9.07	50.7 ± 8.92
Diet quality score, points	7.36 ± 1.02	7.03 ± 0.93	6.03 ± 1.02	5.13 ± 0.82	5.00 ± 0.72	4.16 ± 0.73
BMI at baseline, kg/m^2^	26.4 ± 4.23	26.3 ± 4.26	26.2 ± 4.29	26.2 ± 4.24	26.3 ± 4.41	26.2 ± 4.25
BMI at FUP3, kg/m^2^	26.8 ± 4.32	26.6 ± 4.28	26.5 ± 4.33	26.6 ± 4.36	26.6 ± 4.48	26.5 ± 4.27
Physical activity at baseline, h/week	1.50 (4.00)	2.00 (4.00)	1.50 (4.00)	2.00 (4.00)	2.00 (4.00)	1.50 (4.00)
Physical activity at FUP2, h/week	2.00 (4.25)	2.25 (4.00)	2.00 (4.25)	2.00 (4.00)	2.00 (4.50)	2.00 (4.25)
Education, % university degree	41.0	40.9	35.7	40.6	39.2	36.6
Occupation at baseline, % employed	72.4	71.8	67.4	71.0	68.7	68.9
Occupation at FUP3, % employed	55.6	57.4	53.3	58.4	55.2	53.5
Total energy intake at baseline, kcal/day	2275 ± 738	2175 ± 669	2052 ± 655	2263 ± 724	2137 ± 661	2080 ± 668
Total energy intake at FUP3, kcal/day	2264 ± 787	2194 ± 697	2127 ± 615	2318 ± 798	2187 ± 679	2127 ± 628
Smoking status at baseline, % current smoker	21.7	19.4	18.8	21.8	17.1	19.5
Smoking status at FUP3, % current smoker	17.2	15.3	14.2	18.0	13.3	14.7
Alcohol consumption at baseline (g/d)	17.6 ± 25.1	15.1 ± 22.9	13.0 ± 21.8	17.4 ± 29.4	13.4 ± 18.4	13.7 ± 21.8
Alcohol consumption at FUP3 (g/d)	17.4 ± 22.3	15.0 ± 20.0	12.9 ± 19.3	17.1 ± 25.6	13.4 ± 16.7	13.6 ± 19.3
Change in diet in last 2 years before FUP3, % yes or partly	39.9	44.3	46.3	39.8	43.4	46.3

Characteristics presented as percentages, mean ± standard deviation or median (interquartile range); FUP3—follow-up 3; Change in diet quality scores reflects relative change from baseline to follow-up 3 (median time difference: 6.6 years).

### Associations of changes in HDS with chronic diseases

Higher adherence to the baseline HDS resulted in both significant lower T2D risk (HR_Q5 vs Q1_ = 0.82; 95% CI 0.69–0.97; HR per 1 SD = 0.90; 95% CI 0.85–0.96) and lower stroke risk (HR_Q5 vs Q1_ = 0.57; 95% CI 0.40–0.82; HR per 1 SD = 0.83; 95% CI 0.73–0.94) over a mean follow-up of 11 years (Supplementary Table 6).

The analysis of the change in HDS did not result in significant associations for the three chronic disease outcomes when the change was modelled continuously (Table 2). The RCS analysis did not indicate any deviation from linearity (Supplementary Fig. 2A,C,E). Still, when comparing categories of change, participants who decreased their HDS had higher risk of stroke (HR 1.80; 95% CI 1.02–3.20) compared to participants with stable HDS (Table 2).

**Table 2:** Hazard Ratio and 95% confidence intervals for the association of diet quality score changes with chronic disease risk (mean follow-up time = 4.7 years).

Outcome	Diet score		Decrease (> 5%)	Stable (± ≤ 5%)	Increase (> 5%)	Per 20% increase in diet score
Type 2 diabetes	Mediterranean Pyramid	Cases/Person-years	114/16,349	115/17,497	339/60,663	
		Model 1	1.05 (0.81–1.37)	1.00	0.71 (0.57–0.90)	0.93 (0.84–1.02)
		Model 2	1.03 (0.79–1.33)	1.00	0.74 (0.59–0.92)	0.94 (0.85–1.03)
	Healthy Diet Score	Cases/Person-years	79/13,703	86/14,061	403/66,745	
		Model 1	0.94 (0.69–1.28)	1.00	0.82 (0.64–1.05)	0.94 (0.86–1.03)
		Model 2	0.95 (0.70–1.30)	1.00	0.89 (0.69–1.14)	0.96 (0.88–1.05)
Myocardial infarction	Mediterranean Pyramid	Cases/Person-years	34/17,828	30/18,830	107/65,445	
		Model 1	1.02 (0.62–1.68)	1.00	0.98 (0.64–1.51)	0.97 (0.82–1.15)
		Model 2	1.02 (0.62–1.67)	1.00	0.98 (0.64–1.51)	0.97 (0.82–1.15)
	Healthy Diet Score	Cases/Person-years	29/14,583	23/15,230	119/72,290	
		Model 1	1.20 (0.69–2.08)	1.00	0.97 (0.60–1.56)	1.01 (0.86–1.17)
		Model 2	1.19 (0.69–2.08)	1.00	0.98 (0.61–1.58)	1.01 (0.87–1.17)
Stroke	Mediterranean Pyramid	Cases/Person-years	44/17,861	26/19,021	119/65,734	
		Model 1	1.67 (1.02–2.72)	1.00	1.23 (0.78–1.92)	0.89 (0.75–1.05)
		Model 2	1.67 (1.02–2.72)	1.00	1.23 (0.79–1.92)	0.89 (0.75–1.05)
	Healthy Diet Score	Cases/Person-years	31/14,717	19/15,302	139/72,597	
		Model 1	1.80 (1.01–3.20)	1.00	1.15 (0.69–1.91)	0.92 (0.79–1.06)
		Model 2	1.80 (1.02–3.20)	1.00	1.15 (0.69–1.91)	0.92 (0.79–1.06)

Model 1: adjusted for sex, baseline diet quality score and baseline education, baseline and FUP3 information on: occupation, smoking status, self-reported hypertension, lifetime alcohol consumption, total energy intake, and physical activity (FUP2); Model 2: Model 1 + baseline and FUP3 BMI; FUP-3—follow-up 3; Change in diet quality scores reflects relative change from baseline to follow-up 3 (median time difference: 6.6 year).

Interaction analyses did not support that associations differed between men and women (all p for interaction > 0.05) and we did not observe any significant associations in stratified analyses by sex (Supplementary Table 7).

The stratified analyses by baseline HDS tertiles did not indicate significant associations for the 20% increase in HDS with incident T2D and stroke, but a significant lower MI risk (HR 0.66; 95% CI 0.49–0.91) in those participants starting in the “medium” HDS tertile at baseline (Supplementary Table 8). Still, none of the interaction terms were statistically significant (all p for interaction > 0.05). Since the RCS analysis indicated non-linear associations for stroke risk in the “low” and “high” HDS baseline tertile (Supplementary Fig. 3), additional analyses for the comparison of change categories were conducted. Starting with “low” HDS tertile at baseline, an increase in HDS compared to the stable reference pointed towards a lower stroke risk (HR 0.48; 95% CI 0.21–1.13). Starting in the “high” HDS tertile at baseline and decrease the HDS compared to the stable category pointed towards a higher stroke risk (HR 2.29; 95% CI 0.93–5.66) (data not shown).

The sensitivity analyses on the exclusion of participants with implausible energy intake or those, who reported a change in diet due to weight gain, blood pressure, dyslipidemia or diabetes diagnosis did not indicate altered associations between a longitudinal HDS change and the three chronic disease outcomes compared to the main results (Supplementary Table 9).

### Associations of changes in MedPyr with chronic diseases

The RCS analysis did not show any deviation from linearity for the association between the change in MedPyr and the three outcomes of interest (Supplementary Fig. 2B,D,F). A 20% increase in MedPyr, modelled continuously, pointed towards a lower T2D risk (HR 0.94; 95% CI 0.85–1.03) over a median follow-up time of 4.7 years (Table 2). When comparing participants in the increase-category to the stable-category as reference, they had a lower T2D risk in Model 1 and this association was not altered by the adjustment for BMI in Model 2 (HR 0.74; 95% CI 0.59–0.92). Participants in the decrease-category had similar risk for T2D in comparison to participants in the stable-category. Longitudinal changes in the MedPyr were not significantly associated with the incidence of MI, neither when modelling changes on a continuous scale, nor when using categories. For the association of a longitudinal MedPyr change with stroke, the analysis per 20% increase in MedPyr pointed towards an inverse association (HR 0.89; 95% CI 0.75–1.05), and the comparison of change categories indicated for participants in the decrease-category compared to the stable reference a significant higher stroke risk (HR 1.67; 95% CI 1.02–2.72).

The stratification of the analysis by sex did not indicate significant differences in the associations of MedPyr change with MI and stroke, but pointed towards a lower T2D risk for the 20% increase in MedPyr in women (HR 0.89; 95% CI 0.77–1.04) (Supplementary Table 7). Still, none of the interaction terms reached statistical significance.

We next evaluated if associations of changes in MedPyr with cardiometabolic endpoints depend on baseline adherence to this diet. The interaction terms did not reach statistical significance (Supplementary Table 8). Still noteworthy, the stratification by baseline MedPyr tertiles pointed towards a lower risk for all three outcomes per 20% increase in MedPyr in those participants, who started in the “medium” MedPyr tertile at baseline. RCS analysis indicated a non-linear association with incident T2D in the “low” baseline MedPyr tertile (Supplementary Fig. 4). Here, the comparison of change categories pointed towards a lower T2D risk (HR 0.78; 95% CI 0.48–1.28) for an increase in MedPyr compared to the stable category and towards a higher T2D risk (HR 1.47; 95% CI 0.78–2.78) for those, who decreased MedPyr compared to the stable reference (data not shown). The RCS analysis did not indicate deviations from linearity across the baseline MedPyr tertiles for associations with MI and stroke (Supplementary Fig. 4).

In a sensitivity analysis, the removal of the alcohol component from MedPyr resulted in stronger inverse associations of MedPyr with incident T2D (HR per 20% increase = 0.90; 95% CI 0.81–1.00) and stroke (HR per 20% increase = 0.83; 95% CI 0.70–1.00) (Supplementary Table 9). The exclusion of participants with implausible total energy intake, and the exclusion of those, who reported either a change in diet due to weight gain, high blood pressure, dyslipidemia or diabetes, did not change the associations with T2D, MI and stroke compared to the main analysis (Supplementary Table 9).

## Discussion

Repeated assessments of diet in the EPIC-Potsdam study allowed to quantify longitudinal change in diet quality scores adherence over a period of approximately 7 years and we observed that participants, who increased their MedPyr by more than 5% had a significantly lower risk of T2D compared to participants with no changes in MedPyr. This inverse association appeared to be more pronounced in women and those participants starting with a low and medium adherence to MedPyr at baseline. A decrease in MedPyr adherence by more than 5% was associated with a higher risk for stroke, while changes in MedPyr score adherence were not associated with MI risk. A higher adherence to the HDS at baseline was associated with a lower risk for T2D and stroke. Longitudinal changes of the HDS were not clearly associated with T2D and MI risk, but a decrease in HDS adherence by more than 5% was associated with a higher stroke risk in our study population.

So far, we could only identify two studies that have investigated longitudinal changes in diet quality scores in relation to cardiometabolic disease risk: In the U.S., one study investigated the association between AHEI changes and T2D risk and concluded for a > 10% increase in AHEI over 4 years a lower T2D risk^12^, but no study has yet investigated the association with a change in a diet quality score reflecting Mediterranean diet with regards to T2D risk. However, the association of changes in several dietary quality scores, including AHEI, aMED and DASH with CVD risk were investigated in three U.S. cohorts^13^. The investigators concluded no significant association for an increase in aMED with total CVD risk, but a lower risk for stroke. However, a comparison to our results is limited due to the different composition and calculation of aMED and MedPyr^7,13^: While the MedPyr evaluates absolute intakes of 15 components of a typical healthful Mediterranean diet, the aMED is restricted to the evaluation of relative intakes of 9 components, leaving out dairy products, eggs, white meat, sweets and potatoes, and considering the ratio of monounsaturated to saturated fatty acids instead of the consumption of olive oil. A study in England (EPIC-Norfolk) investigated different Mediterranean diet scores and their associations with CVD outcomes by using repeated dietary data and showed inverse associations between diet scores which considered absolute intakes (e.g. MedPyr) and CVD, ischaemic heart disease and stroke^9^. However, comparability to our results is constrained, because cumulative averages were calculated instead of changes in diet quality scores.

Our results on changes in MedPyr and risk of T2D extent our previous investigation on adherence to baseline MedPyr^7^, where a higher adherence to a Mediterranean diet was related to lower risk of T2D. Our current analyses support that changes of dietary intake towards a Mediterranean diet over time can reduce T2D risk. Contrary to previous reported attenuated associations with T2D after exclusion of the alcohol component from a Mediterranean diet score in the EPIC-InterAct consortium^31^, our results indicated an enhanced lower risk of T2D. Since the participants in our study have not changed their alcohol consumption over time, the exclusion of this component could in our particular case enhance precision of the model which could explain the result of this sensitivity analysis. Although adherence to baseline MedPyr was not associated with stroke risk in our cohort^7^, a decrease in MedPyr over time, in comparison to participants with a stable MedPyr, pointed towards a higher risk for stroke. This underpins the added value of this change analysis. Interestingly, the inverse association of MedPyr with stroke was more pronounced after exclusion of the alcohol component. This is unexpected, given that a systematic review including 27 cohorts with 1.4 million participants concluded a J-shaped association for alcohol intake and stroke morbidity^32^.

A recently published investigation of 8 different diet quality indices with major chronic diseases in three U.S. cohorts concluded for 6 commonly used indices reflecting dietary guidelines or regional dietary habits, e.g. AHEI, DASH or aMED, significant disease risk reductions^33^. Comparable to an index like the AHEI, the components for the HDS in our investigation were also selected based on the national dietary guidelines in Germany and evidence from single food associations with T2D, CVD and cancer^23^. Similarly, we observed significant risk reductions for T2D and stroke in our study population, comparing extreme quintiles of baseline adherence to the HDS. Wang et al. also investigated two empirically-derived mechanism-based indices using components with hyperinsulinemic and inflammatory properties^34^ and showed the strongest associations with disease risk reduction in the three investigated U.S. populations^33^. Although our analysis was restricted to only two guideline-based diet quality scores, we contributed new insights into the associations between longitudinal changes of these with the T2D, MI and stroke risk. Interestingly, although only a decrease in HDS adherence was associated with a significant higher stroke risk in the main analysis, the stratified analysis by baseline HDS tertile pointed towards a reduction in stroke risk when participants increased HDS adherence at least in the “low” HDS tertile. Similarly, Sotos-Prieto et al. observed for 4-year changes in aMED and DASH a reduced risk for stroke^13^. Several methodological constraints potentially explained the null associations between changes in HDS with T2D and MI risk in our study: One aspect in evaluating dietary changes in cohort studies is random measurement error, which may result in misclassification of individuals (e.g. false assignment to categories of decreased or increased diet quality score) and would most likely tend to attenuate associations. Different degrees of measurement error for different diet quality scores may be an explanation for the null associations between the HDS and chronic disease outcomes in comparison to the MedPyr. This notion is supported by differences in reliability measures, which we observed in a sub study in EPIC-Potsdam using repeated FFQs, filled out by participants one year apart. For the HDS, reliability was moderate (r = 0.53) but lower than what we have observed for the MedPyr (r = 0.62)^23,24^. A reason could be the low reliability of some components, which are part of the HDS, but not of the MedPyr. So, for example, the reliability of the aggregated food group “vegetable oils”, included in the HDS, was very low (r = 0.07), while olive oil, as component of the MedPyr, had a moderate reliability (r = 0.54).

A more general limitation is the relatively short follow-up time of 4.4 years in our study, because we only considered incident cases occurring after the actual change in the two diet quality scores (after FUP3). The small number of incident cases may have limited statistical power, in particular for stratified and interaction analyses, and resulted in relative imprecise estimates. However, a strength of the outcome ascertainment in our study was the very low misclassification rate of cases (T2D: 0.25%; MI: 0.15%, Stroke: 0.07) and the two-step approach of using different sources like self-report, medication or reasons to change the diet to firstly identify potential disease cases (Supplementary Table 10), but a mandatory second step of verification by the treating physician. The few non-verified cases were excluded from this analysis, but we would not expect a major change in results except for a slight improvement in precision of the estimates due to a higher statistical power. We could not rule out that differences in the structure of the two applied FFQs at baseline and FUP3 had an impact on the calculated longitudinal change in diet quality scores. However, an important initial step of our analysis was the evaluation of dietary data from a comparison study (n = 512) to identify systematic differences between the two FFQs and to correct food groups for such differences. Up to now, consequences of changes in the structure of dietary assessment instruments in longitudinal studies to capture certain trends of dietary intake, and its impact on disease-associations, have not been addressed systematically, but should get more attention in future studies.

As strengths of our analyses, we were able to consider dietary data not only from one time point, but additionally from a second time point in a large sample of more than 23,000 participants. Even more time points could have enabled the modeling of the exposure as time-varying change in diet score, as it was conducted in two U.S. studies^12,13^. This was not possible in our analysis. Similar to another U.S. study, which investigated how change in DASH diet was associated with cardiovascular risk factors in young people with diagnosed diabetes^35^, we considered not only the change in diet scores but also adjusted for the baseline diet quality score in our models. Furthermore, similar to an investigation in the ARIC study^25^, we have compared the risk to develop T2D, MI or stroke in categories of increased/decreased diet scores in comparison to a stable reference category. Another approach to consider repeated measures of diet scores, which was reported in several studies^9,11,15^, was the calculation of cumulative averages, if at least two time points of dietary measurement were available. Nevertheless, this would not be the ideal approach to detect changes in dietary intake over time as we intended. The restriction to the two diet quality scores MedPyr and HDS in our study could be a limitation, since there is current evidence on many more diet quality scores being associated with the cardiometabolic diseases of interest^3,25,33^. We restricted the analyses to the MedPyr to follow-up on the previous investigation in our EPIC-Potsdam study: baseline MedPyr adherence was associated with lower T2D risk in the total population and lower MI risk in women^7^. We aimed to investigate, if a change towards a higher MedPyr score would be also beneficial in terms of these health outcomes. The focus on the HDS had several reasons: (1) the aforementioned gap in terms of investigations on disease associations, (2) the consideration of absolute intake ranges comparable to MedPyr, (3) the investigation of a diet score which includes German dietary recommendations as an alternative approach to a Mediterranean-originated diet. Besides data on dietary intake, several data on socio-demographics and lifestyle factors were collected with the self-administered questionnaire at FUP3. Hence, we were able to adjust the regression models for several potential confounders on the diet-disease-associations like age, sex, education and occupation, physical activity, smoking and alcohol consumption and considered both data from baseline and FUP3. Furthermore, we adjusted for self-reported hypertension, since knowledge about this health condition could have led to a higher awareness for a healthy lifestyle and subsequently to a change in dietary behavior. To rule out that the association was mainly driven by total energy intake instead of diet composition, the regression models were adjusted for it at baseline and FUP3. We adjusted the models additionally for the baseline diet quality score to consider the so-called “exogenous change”, which means all non-random changes in the diet quality score at FUP3 which were not predetermined by the diet quality score at baseline^36^. Since BMI could theoretically be a confounder or on the mediating path to the chronic diseases of interest, we decided to adjust for BMI at baseline and FUP3 in a separate model. No major changes in the HRs were observed. Nevertheless, we cannot fully rule out that residual confounding could be still existent.

A further strength was the investigation of potential effect measure modifiers like sex^29,30^ and baseline diet score. Although the non-significant statistical interactions would not support any sex differences in associations, our observation of a reduced T2D risk with increased MedPyr might be restricted to women supports sex-stratified analyses. Similar to our observation, Wang et al. also observed stronger associations for the investigated DQIs in women than in men^33^. Furthermore, although the statistical interaction with baseline diet score tertiles was not significant, the results observed in the stratified analyses supported the findings from the main analyses and gave insights beyond that. A further strength was the conduct of more sensitivity analyses, e.g. to eliminate the possibility of reverse causation. To do so, we excluded those participants, who reported a change in diet due to weight gain, high blood pressure, dyslipidemia or diabetes. Since the results were not changed, they indicated robust associations. Nevertheless, we could not rule out that there were other unmeasured confounders (e.g. subclinical medical conditions), which could have led to a change in dietary behavior and were associated with the investigated chronic diseases.

## Conclusion

In this middle-aged population of the EPIC-Potsdam study, an increase in adherence to a Mediterranean Diet, measured with the MedPyr, was associated with a lower T2D risk. While changes in MedPyr were not appreciably related to MI risk, a decrease in MedPyr adherence was related to higher stroke risk. The findings contribute further evidence on advantages of an actual change in dietary behavior towards a Mediterranean Diet. While higher adherence in HDS at baseline was related to lower risk of T2D and stroke, a decrease in HDS adherence over time was associated with a higher stroke risk, but changes in HDS were not associated with T2D and MI risk.

### Supplementary Information

Supplementary Information.

## Data Availability

The datasets generated and/or analysed during the current study are not publicly available due to German Federal and State data protection regulations, but are available from the secretariat of the Human Study Center (Office.HSZ@dife.de) on reasonable request.
