# Supplementary material for: Longitudinally changed diet quality scores and their association with type 2 diabetes mellitus and cardiovascular diseases in the EPIC-Potsdam study
Source: Sci Rep. 2024 Jun 17;14:13907. doi: 10.1038/s41598-024-63899-8 (PMC11183239; doi:10.1038/s41598-024-63899-8)
Supplement: Supplementary file 1 — Supplementary Information. [file 41598_2024_63899_MOESM1_ESM.docx]

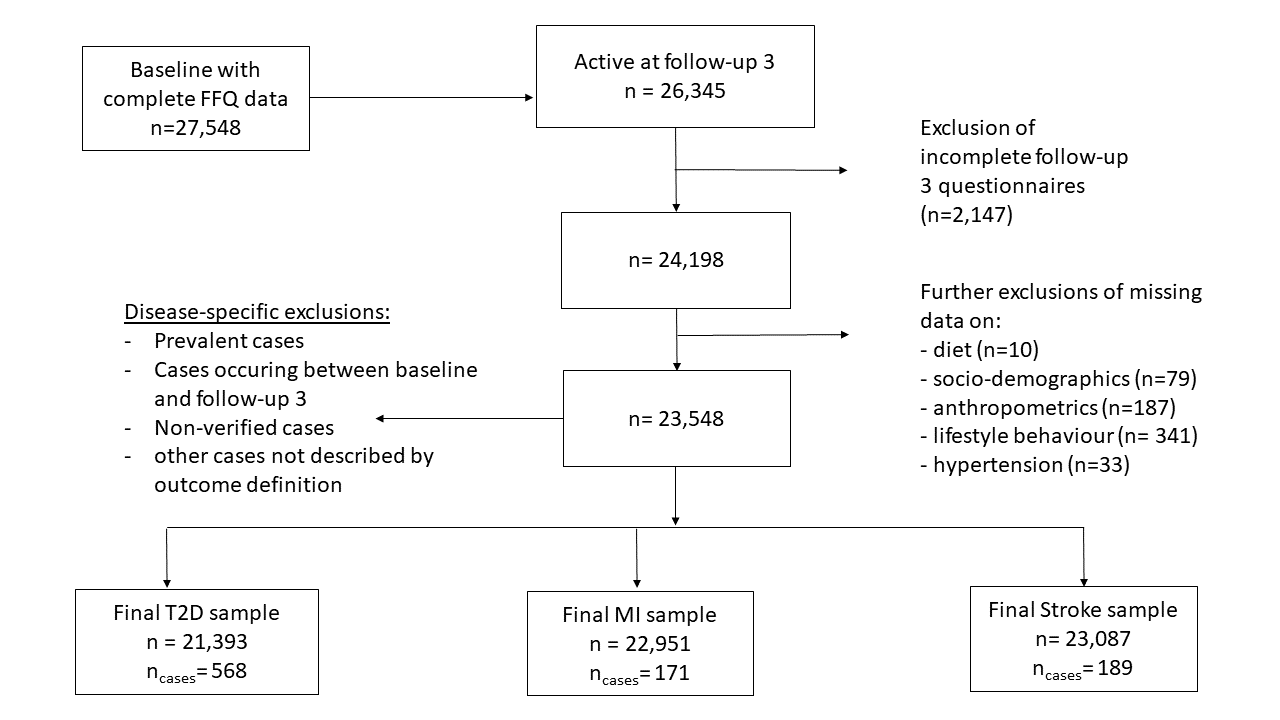


**Supplementary Figure 1 Flow chart of the included number of participants for the disease specific analyses**

FFQ – food frequency questionnaire; MI – myocardial infarction; T2D – type 2 diabetes


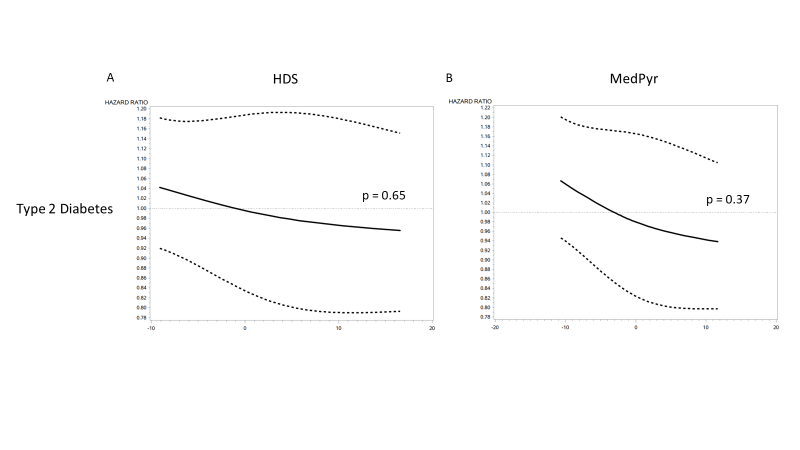

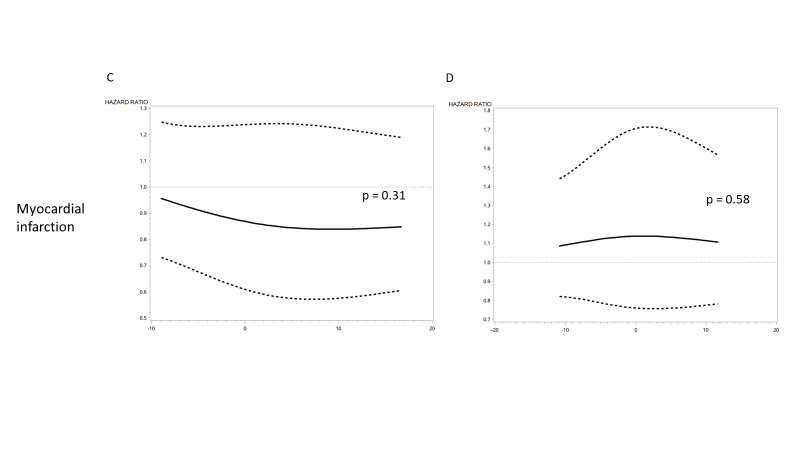

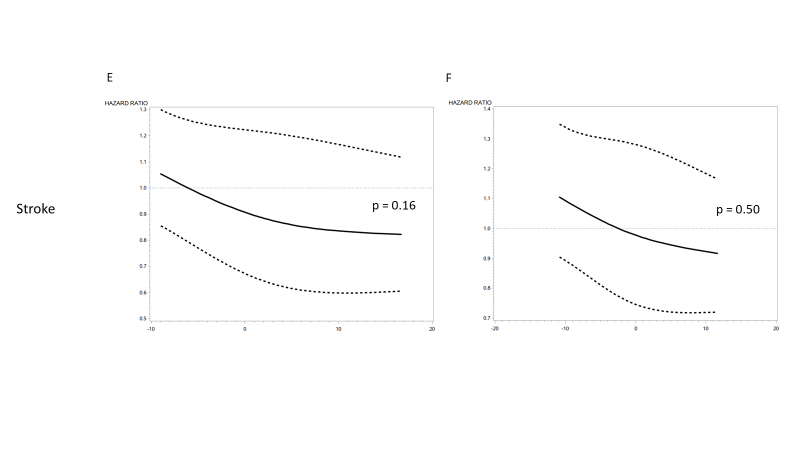


**Supplementary Figure 2 Restricted cubic splines (RCS) for the changes in MedPyr and HDS and their associations with T2D, MI and stroke**

**A** RCS for the association between HDS change and T2D; **B** RCS for the association between MedPyr change and T2D; **C** RCS for the association between HDS change and MI; **D** RCS for the association between MedPyr change and MI; **E** RCS for the association between HDS change and stroke; **F** RCS for the association between MedPyr change and stroke.


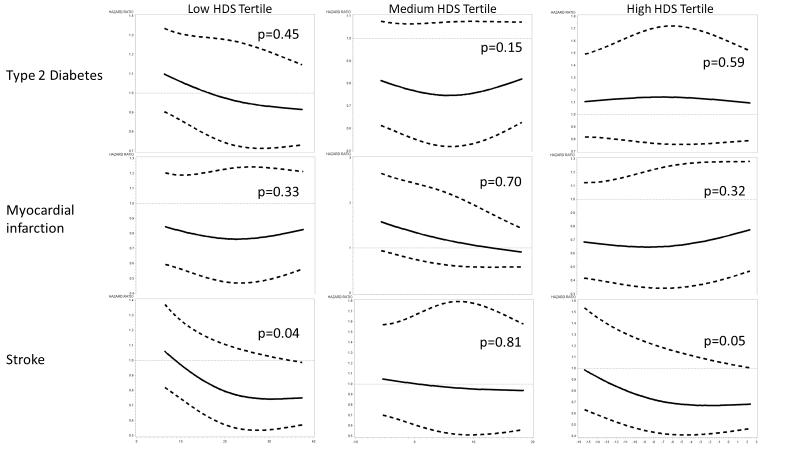


**Supplementary Figure 3 Restricted cubic splines (RCS) for the association between change in HDS and T2D, MI and stroke in tertiles of baseline HDS**


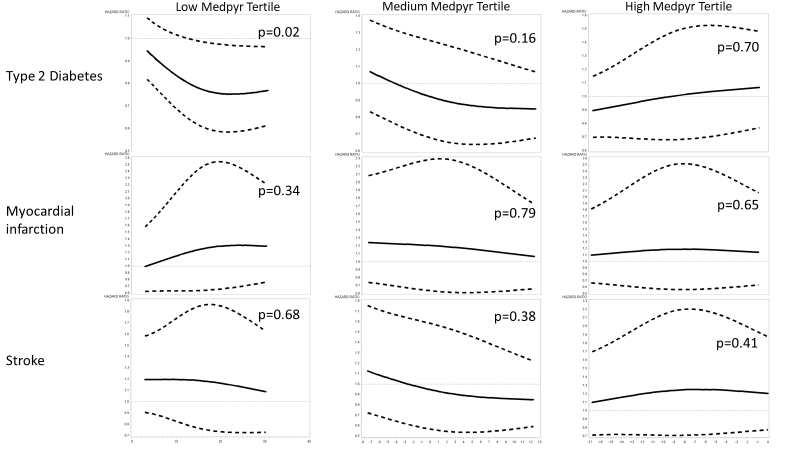


**Supplementary Figure 4 Restricted cubic splines (RCS) for the association between change in MedPyr and T2D, MI and stroke in tertiles of baseline MedPyr**

**Supplementary Methods**

Systematic investigation of differences between FFQ_0_ and FFQ_1_

To address systematic differences between the two FFQs, we used data from a comparison study (n = 512) which was conducted within the FUP3 of EPIC-Potsdam. Participants, who volunteered to take part, were mailed the FFQ_0_ on average 28 days after they have filled in the FFQ_1_. These participants had comparable characteristics to the overall cohort with regards to the distribution of gender, age, BMI, smoking behavior and educational level [1]. For the purpose of investigating the diet quality scores, we derived 44 congruent food groups from both dietary assessment instruments, where respective food items were condensed as shown in **Supplementary Table 1**. For the majority of food groups, the EPIC-Soft food classification system was adopted. It consists of 17 main food groups and more details were provided by two additional layers of subgroups [2]. For some exceptions, specific food items were condensed into food groups, when the EPIC-Soft classification did not provide enough granularity. Subsequently, sex-specific median and interquartile range for the intake of each food group was calculated in the comparison study for both FFQs. Furthermore, the sex-specific proportion of FFQ_1_ in FFQ_0_ food group median intake was estimated, which was subsequently used as correction factor. The correction factors for each of the 44 food groups, derived in the comparison study, were then multiplied with the FFQ_1_ food group intake in the full EPIC-Potsdam cohort to correct for systematic differences, which occurred due to changes in the assessment instrument design.

**Supplementary Table 1 List of harmonized food groups and their condensed food items at baseline and FUP3 in the EPIC-Potsdam study**

| Number of food group | Food group | Condensed food items |
| --- | --- | --- |
| 1 | Bread | Group 06 03: Bread, crispbread, rusks |
| 2 | Whole-Grain Bread | foodk=1010 (wholemeal bread)  foodk=1011 (wholemeal roll)  foodk=1050 (dark- and wholemeal roll) |
| 3 | Cereals | Group 06 04: Breakfast cereals |
| 4 | Pasta | Group 06 02: Pasta, rice, other grain |
| 5 | Cakes and Cookies | Group 12: Cakes |
| 6 | Confect | Group 11 02: Chocolate, candy bars, paste |
| 7 | Desserts | Group 05 06: Cream desserts, puddings  Group 11 05: Ice cream, water ice |
| 8 | Sweet spreads | Group 11 01: Sugar, honey, jam |
| 9 | Eggs | Group 09: Egg and egg products |
| 10 | Fresh fruits | Group 04 01: Fruits |
| 11 | Other fruits | Group 04 03: Mixed fruits  Group 04 04: Olives |
| 12 | Nuts | 04 02: Nuts and seeds |
| 13 | Leafy vegetables | 02 01: Leafy vegetables |
| 14 | Fruiting vegetables | 02 02: Fruiting vegetables |
| 15 | Root vegetables | 02 03: Root vegetables |
| 16 | Cabbage | 02 04: Cabbages |
| 17 | Other vegetables | 02 05: Mushrooms  02 06: Grain and pod vegetables  02 07: Onion, garlic  02 08: Stalk vegetables, sprouts  02 09: Mixed salad, mixed vegetables |
| 18 | Legumes | 03: Legumes |
| 19 | Potatoes | 01: Potatoes and other tuber |
| 20 | Fermented dairy | 05 03: Yogurt |
| 21 | Non-fermented dairy | 05 01: Milk  05 02: Milk beverages  05 07 01: Dairy creams  05 08: Milk for coffee and creamers |
| 22 | Cheese | 05 04: Fromage blanc, petits suisses  05 05: Cheeses |
| 23 | Butter | 10 02: Butter |
| 24 | Margarine | 10 03: Margarines |
| 25 | Olive oil | foodk=19062 (Olive oil to prepare salads)  foodk=19063 (Olive oil to prepare vegetables)  foodk=19065 (Olive oil to prepare meat dishes) |
| 26 | Other vegetable oils | 10 01: Vegetable oils |
| 27 | Other fat | 10 00: unclassified  10 04: deep frying fats  10 06: other animal fat |
| 28 | Other non-alcoholic beverages | 13 03 03: Herbal tea  13 04: Waters |
| 29 | Coffee | 13 03 01: Coffee |
| 30 | Tea | 13 03 02: Tea |
| 31 | Fruit juice | 13 01: Fruit and vegetable juices |
| 32 | Soft drinks | 13 02: Carbonated/soft/isotonic drinks |
| 33 | Beer | 14 03: Beer, cider |
| 34 | Wine | 14 01: Wine |
| 35 | Spirits | 14 04: Spirits, brandy  14 05: Aniseed drinks |
| 36 | Other alcohol | 14 02: Fortified wines  14 06: Liquers  14 07: Cocktails, punches |
| 37 | Fish | 08 01: Fish |
| 38 | Poultry | 07 02: Poultry |
| 39 | Red meat | 07 01: Red meat |
| 40 | Processed meat | 07 04: Processed meat |
| 41 | Offals | 07 05: Offals |
| 42 | Condiments | 15: Condiments and sauces |
| 43 | Soups | 16: Soups, bouillon |
| 44 | Miscellaneous | 17: Miscellaneous |

**Supplementary Table 2 Scoring criteria for the Mediterranean Pyramid (MedPyr) as described by Galbete et al. [3]**

| Component | Recommended Intake | Score of 0 | Score of 1 |
| --- | --- | --- | --- |
| Vegetables | ≥ 6/day | 0/day | ≥ 6/day |
| Legumes | ≥ 2/wk | 0/wk | ≥ 2/wk |
| Fruits | 3 – 6/day | 0/day | 3 – 6/day |
| Nuts | 1 - 2/day | 0/day | 1 - 2/day |
| Cereals | 3 – 6/day | 0/day | 3 – 6/day |
| Dairy | 2/day | 0/day | 2/day |
| Fish | ≥ 2/wk | 0/wk | ≥ 2/wk |
| Red meat | < 2/wk | ≥4/wk | < 2/wk |
| Processed meat | ≤ 1/wk | ≥2/wk | ≤ 1/wk |
| White meat | 2/ wk | 0/wk | 2/ wk |
| Egg | 2 – 4/wk | 0/wk | 2 – 4/wk |
| Potatoes | ≤ 3/wk | ≥6/wk | ≤ 3/wk |
| Sweets | ≤ 2/wk | ≥4/wk | ≤ 2/wk |
| Alcohol | 10 – 50g/day for men, 5-25g/day for women | >50g/day for men, >25g/day for women | 10 – 50g/day for men, 5-25g/day for women |
| Olive oil | Principal source of dietary lipids | Non-consumers | Consumers |

**Supplementary Table 3 Scoring of the Healthy Diet Score (HDS) according to Jannasch et al. [4]**

| Components | Recommended intake | Maximum score | Standard for maximum | Standard for minimum |
| --- | --- | --- | --- | --- |
| Bread and cereals  Overall intake | Moderate intake:  3-5 portions/d | 0.5 points | 3-5 portions/d | 0 < 1 portion |
| Proportion of whole grains | High intake:  100 % | 0.5 points | 100 % | 0 % |
| Fermented dairy products | Moderate intake:  1-2 portions/d | 1 point | 1-2 portions/d | None to < 1 portion  More than 4 portions |
| Raw and cooked vegetables | High intake:  ≥ 3 portions/d | 1 point | ≥ 3 portions/d | None to < 1 portion |
| Fruits | High intake:  ≥ 2 portions/d | 1 point | ≥ 2 portions/d | None to < 1 portion |
| Legumes | High intake:  ≥ 2 portions/w | 1 point | ≥ 2 portions/w | None to < 1 portion |
| Unsalted nuts | Moderate intake:  7 portions/w | 1 point | 7 portions/w | None to < 3 portions |
|  |  |  |  |  |
| Fish  Overall intake | Moderate intake:  2 portions/w | 0.5 points | 2 portions/w | None to < 1 portion |
| Proportion of fatty marine Fish | High intake:  100 % | 0.5 points | 100 % | 0 % |
| Meat  Processed meat | Low intake:  < 1 portion/w | 0.5 points | None to < 1 portion/w | > 2 portions/w |
| Red meat | Low intake:  ≤ 2 portions/w | 0.5 points | None to 2 portions/w | > 4 portions/w |
| Vegetable oils  Overall intake | High intake:  ≥ 7 times/w | 0.5 points | ≥ 7 times/w | None to ≤ 3 times/w |
| General use for food preparation | High intake:  100 % | 0.5 points | 100 % | 0 % |
| Sugar-sweetened beverages | Low intake:  1 glass/w or less | 1 point | None to < 1 glass/w | ≥ 2 glasses/w |

**Supplementary Table 4** **Median intake and interquartile range of all food groups (g/day) assessed at baseline across the change categories of the two diet quality indices**

| **Food groups (g/day) at baseline** | **Change in Mediterranean Pyramid** | | | **Change in Healthy Diet Score** | | |
| --- | --- | --- | --- | --- | --- | --- |
|  | **Decrease (>5%)** | **Stable (± ≤5%)** | **Increase (>5%)** | **Decrease (>5%)** | **Stable (± ≤ 5%)** | **Increase (>5%)** |
| Bread | 186 (94.6) | 177 (94.5) | 159 (96.0) | 180 (88.6) | 172 (86.0) | 163 (99.5) |
| Whole-grain bread | 26.4 (66.8) | 30.4 (64.8) | 23.4 (55.0) | 34.3 (78.2) | 38.0 (73.9) | 21.9 (51.6) |
| Cereals | 0.28 (4.78) | 0.55 (5.70) | 0.55 (4.78) | 0.55 (5.24) | 0.60 (6.04) | 0.33 (4.78) |
| Pasta | 15.1 (17.2) | 14.3 (16.1) | 13.9 (15.6) | 15.4 (17.4) | 15.1 (16.5) | 13.6 (15.6) |
| Cakes and Cookies | 44.0 (63.8) | 45.7 (60.2) | 46.2 (56.1) | 48.2 (62.3) | 46.2 (59.7) | 45.2 (56.7) |
| Confect | 6.45 (13.0) | 6.67 (12.9) | 6.61 (12.1) | 7.12 (13.5) | 6.45 (12.8) | 6.45 (12.0) |
| Desserts | 1.79 (5.19) | 1.81 (5.19) | 1.84 (5.18) | 2.64 (5.18) | 1.84 (5.18) | 1.79 (5.19) |
| Sweet spreads | 10.8 (20.1) | 10.8 (18.4) | 10.9 (18.3) | 10.8 (19.2) | 10.8 (18.0) | 10.9 (18.8) |
| Eggs | 16.8 (14.8) | 16.4 (14.8) | 12.9 (13.4) | 14.2 (13.2) | 13.4 (13.2) | 13.4 (12.8) |
| Fresh fruits | 158 (140) | 144 (121) | 120 (104) | 186 (155) | 184 (139) | 111 (101) |
| Other fruits | 2.54 (3.54) | 2.39 (3.33) | 2.31 (3.11) | 2.61 (3.87) | 2.39 (3.49) | 2.29 (2.99) |
| Nuts | 1.40 (3.73) | 1.41 (3.53) | 0.85 (3.12) | 1.40 (3.61) | 1.42 (3.49) | 0.86 (3.13) |
| Leafy vegetables | 6.81 (8.84) | 7.18 (8.18) | 6.56 (8.00) | 8.61 (11.1) | 8.52 (10.2) | 6.12 (7.17) |
| Fruiting vegetables | 58.1 (42.8) | 57.3 (39.5) | 53.3 (36.8) | 69.2 (55.1) | 66.9 (45.6) | 50.5 (33.8) |
| Root vegetables | 11.7 (12.5) | 11.5 (11.0) | 11.3 (10.7) | 13.6 (14.1) | 13.5 (13.2) | 10.6 (9.88) |
| Cabbage | 14.4 (15.1) | 14.3 (13.9) | 14.4 (14.4) | 16.1 (16.6) | 16.2 (15.8) | 13.8 (13.8) |
| Other vegetables | 24.1 (17.5) | 23.0 (16.6) | 22.5 (15.9) | 26.1 (19.0) | 25.3 (17.7) | 21.9 (15.2) |
| Legumes | 1.90 (4.29) | 1.56 (3.83) | 1.42 (3.55) | 1.87 (4.48) | 1.53 (3.92) | 1.44 (3.55) |
| Potatoes | 78.8 (61.5) | 80.1 (65.3) | 94.6 (72.6) | 93.1 (73.4) | 88.9 (67.1) | 88.0 (70.6) |
| Fermented dairy | 53.4 (94.6) | 53.6 (94.5) | 53.4 (94.6) | 53.4 (94.4) | 53.5 (93.7) | 53.4 (94.7) |
| Non-fermented dairy | 59.36 (129) | 63.92 (132) | 59.76 (138) | 63.0 (137) | 60.6 (136) | 59.6 (135) |
| Cheese | 52.20 (42.4) | 50.96 (40.9) | 48.68 (43.2) | 47.8 (31.4) | 49.2 (33.6) | 50.4 (47.4) |
| Butter | 3.85 (14.7) | 3.67 (12.2) | 2.84 (10.3) | 2.40 (10.5) | 2.47 (10.1) | 3.60 (10.9) |
| Margarine | 12.29 (19.6) | 12.29 (18.2) | 12.56 (17.5) | 13.0 (18.2) | 12.7 (17.7) | 12.3 (18.1) |
| Olive oil | 0.72 (1.89) | 0.69 (1.94) | 0.07 (1.17) | 0.62 (2.66) | 0.65 (2.35) | 0.24 (1.20) |
| Other vegetable oils | 3.74 (3.82) | 3.80 (3.50) | 3.31 (3.38) | 4.86 (5.03) | 4.66 (4.49) | 3.12 (2.97) |
| Other fat | 0.28 (0.95) | 0.28 (0.97) | 0.24 (0.84) | 0.27 (0.91) | 0.24 (0.82) | 0.25 (0.89) |
| Other non-alcoholic beverages | 450 (602) | 441 (603) | 441 (586) | 465 (645) | 528 (623) | 422 (572) |
| Coffee | 435 (310) | 406 (310) | 406 (310) | 435 (310) | 406 (310) | 406 (3109 |
| Tea | 24.7 (148) | 23.9 (146) | 21.4 (106) | 23.9 (146) | 24.6 (148) | 21.4 (131) |
| Fruit juice | 124 (224) | 132 (218) | 124 (217) | 131 (236) | 131 (224) | 123 (214) |
| Soft drinks | 1.31 (26.4) | 0.00 (21.2) | 0.00 (19.1) | 1.35 (18.2) | 0.00 (10.4) | 0.81 (25.7) |
| Beer | 71.2 (352) | 41.1 (178) | 20.6 (176) | 41.1 (210) | 22.6 (174) | 35.6 (174) |
| Wine | 42.3 (80.0) | 41.1 (77.8) | 24.7 (60.0) | 32.6 (70.7) | 37.0 (79.5) | 28.8 (65.5) |
| Spirits | 0.33 (2.85) | 0.33 (2.85) | 0.33 (1.64) | 0.33 (1.64) | 0.33 (1.64) | 0.33 (1.64) |
| Other alcohol | 0.41 (0.82) | 0.41 (0.82) | 0.41 (0.82) | 0.41 (0.82) | 0.41 (0.82) | 0.41 (0.82) |
| Fish | 17.6 (19.5) | 17.0 (17.6) | 13.8 (15.0) | 17.6 (19.1) | 17.0 (19.1) | 13.8 (13.8) |
| Poultry | 11.7 (15.5) | 10.1 (13.0) | 8.09 (10.2) | 9.94 (12.8) | 9.56 (12.2) | 8.61 (11.3) |
| Red meat | 26.2 (22.4) | 24.5 (20.9) | 24.0 (21.6) | 26.5 (23.7) | 24.3 (21.6) | 24.2 (21.2) |
| Processed meat | 64.9 (59.0) | 60.0 (50.9) | 57.4 (46.5) | 63.6 (56.0) | 57.5 (47.7) | 58.6 (48.6) |
| Offals | 1.13 (2.19) | 1.11 (2.05) | 1.10 (2.05) | 1.13 (2.08) | 1.09 (2.05) | 1.10 (2.07) |
| Condiments | 13.3 (14.6) | 13.1 (14.7) | 12.5 (13.7) | 14.4 (15.8) | 13.3 (14.8) | 12.3 (13.5) |
| Soups | 32.6 (32.6) | 31.4 (30.5) | 29.8 (28.7) | 33.1 (33.3) | 33.3 (30.7) | 29.5 (28.8) |
| Miscellaneous | 0.23 (0.55) | 0.23 (0.54) | 0.21 (0.52) | 0.23 (0.56) | 0.26 (0.78) | 0.21 (0.49) |

**Supplementary Table 5 Median intake and interquartile range for all food groups (g/day) assessed at FUP3 across the change categories of the two diet quality indices**

| **Food groups (g/day) at FUP3** | **Change in Mediterranean Pyramid** | | | **Change in Healthy Diet Score** | | |
| --- | --- | --- | --- | --- | --- | --- |
|  | **Decrease (>5%)** | **Stable (± ≤5%)** | **Increase (>5%)** | **Decrease (>5%)** | **Stable (± ≤ 5%)** | **Increase (>5%)** |
| Bread | 241 (373) | 210 (281) | 194 (221) | 265 (351) | 228 (258) | 191 (230) |
| Whole-grain bread | 22.9 (36.9) | 23.9 (33.5) | 23.9 (34.8) | 24.6 (38.3) | 26.5 (37.3) | 22.9 (33.1) |
| Cereals | 2.30 (1.28) | 2.30 (1.28) | 2.30 (3.85) | 2.30 (1.28) | 2.30 (3.85) | 2.30 (3.85) |
| Pasta | 21.3 (19.0) | 23.1 (18.9) | 30.6 (18.8) | 21.7 (19.8) | 30.7 (18.8) | 23.3 (18.8) |
| Cakes and Cookies | 51.8 (56.2) | 52.4 (55.3) | 53.9 (53.9) | 57.7 (60.0) | 53.7 (56.1) | 52.4 (54.7) |
| Confect | 8.72 (11.9) | 8.72 (11.9) | 8.72 (11.9) | 11.8 (12.1) | 8.72 (11.9) | 8.72 (11.9) |
| Desserts | 7.15 (7.24) | 7.15 (7.24) | 7.15 (17.2) | 7.15 (16.9) | 7.15 (7.24) | 7.15 (7.24) |
| Sweet spreads | 16.9 (26.4) | 16.9 (25.8) | 16.9 (25.4) | 16.9 (26.3) | 16.9 (25.6) | 16.9 (25.6) |
| Eggs | 15.1 (13.7) | 21.0 (13.3) | 21.0 (12.7) | 21.1 (13.3) | 21.0 (13.0) | 21.0 (12.9) |
| Fresh fruits | 248 (241) | 275 (260) | 331 (266) | 254 (289) | 323 (278) | 309 (259) |
| Other fruits | 0.99 (0.98) | 1.05 (1.11) | 1.10 (1.17) | 0.98 (0.96) | 1.07 (1.10) | 1.09 (1.17) |
| Nuts | 1.25 (2.90) | 1.25 (2.81) | 1.24 (2.81) | 1.25 (2.98) | 1.25 (2.81) | 1.24 (2.79) |
| Leafy vegetables | 10.3 (12.6) | 11.4 (13.0) | 11.6 (13.7) | 8.36 (10.2) | 11.0 (12.6) | 12.1 (14.0) |
| Fruiting vegetables | 85.3 (48.9) | 89.9 (50.8) | 92.5 (54.8) | 76.1 (49.2) | 88.5 (51.9) | 93.8 (53.4) |
| Root vegetables | 20.5 (15.8) | 21.1 (17.5) | 21.6 (18.2) | 19.9 (14.1) | 21.6 (18.2) | 21.5 (17.9) |
| Cabbage | 15.4 (9.07) | 15.3 (9.10) | 15.3 (8.54) | 14.4 (8.75) | 15.2 (8.90) | 15.5 (8.70) |
| Other vegetables | 36.3 (17.0) | 35.5 (16.7) | 35.5 (16.2) | 34.2 (17.1) | 35.1 (16.9) | 36.0 (16.1) |
| Legumes | 1.46 (4.04) | 1.46 (2.92) | 1.46 (2.92) | 1.46 (2.92) | 1.46 (2.92) | 1.46 (2.92) |
| Potatoes | 95.0 (69.3) | 81.5 (59.9) | 67.9 (53.1) | 71.3 (58.6) | 69.4 (57.2) | 78.6 (57.1) |
| Fermented dairy | 22.2 (44.1) | 26.0 (45.8) | 31.2 (50.4) | 23.2 (48.0) | 29.4 (51.8) | 27.6 (46.7) |
| Non-fermented dairy | 60.6 (129) | 70.5 (133) | 73.1 (131) | 66.0 (135) | 74.1 (133) | 70.8 (131) |
| Cheese | 50.6 (38.8) | 52.3 (35.3) | 53.2 (34.1) | 58.0 (49.2) | 55.0 (37.9) | 51.3 (32.5) |
| Butter | 3.76 (8.28) | 3.44 (7.66) | 2.84 (6.63) | 4.23 (9.31) | 3.27 (7.68) | 2.86 (6.57) |
| Margarine | 9.94 (19.0) | 9.90 (15.6) | 10.21 (14.1) | 10.4 (18.1) | 10.1 (15.9) | 10.1 (14.2) |
| Olive oil | 1.50 (2.77) | 1.87 (2.62) | 1.83 (2.71) | 1.11 (1.98) | 1.67 (2.62) | 1.95 (2.80) |
| Other vegetable oils | 5.45 (4.66) | 5.83 (4.64) | 5.81 (4.71) | 4.42 (3.71) | 5.50 (4.43) | 6.10 (4.81) |
| Other fat | 0.18 (0.08) | 0.18 (0.07) | 0.18 (0.07) | 0.18 (0.07) | 0.18 (0.07) | 0.18 (0.07) |
| Other non-alcoholic beverages | 1041 (1193) | 1043 (1148) | 1043 (1132) | 1041 (1207) | 1043 (1132) | 1043 (1148) |
| Coffee | 497 (260) | 493 (218) | 481 (205) | 497 (430) | 478 (254) | 493 (205) |
| Tea | 30.6 (156) | 30.8 (156) | 30.6 (154) | 30.6 (156) | 30.8 (201) | 30.6 (154) |
| Fruit juice | 114 (217) | 129 (230) | 132 (234) | 121 (222) | 130 (239) | 130 (233) |
| Soft drinks | 15.9 (33.9) | 15.9 (33.9) | 15.9 (10.4) | 17.5 (135) | 15.9 (10.4) | 15.9 (10.4) |
| Beer | 35.6 (178) | 20.6 (178) | 12.3 (89.1) | 24.7 (178) | 13.3 (89.1) | 16.4 (143) |
| Wine | 25.2 (67.6) | 31.9 (68.8) | 31.9 (60.7) | 31.1 (66.5) | 31.9 (66.4) | 31.0 (62.2) |
| Spirits | 0.00 (0.98) | 0.00 (0.66) | 0.00 (0.33) | 0.00 (0.82) | 0.00 (0.33) | 0.00 (0.66) |
| Other alcohol | 0.06 (0.55) | 0.12 (0.59) | 0.12 (0.69) | 0.12 (0.75) | 0.12 (0.59) | 0.12 (0.59) |
| Fish | 13.6 (18.5) | 13.6 (22.0) | 13.6 (21.2) | 13.6 (15.3) | 13.6 (14.5) | 13.6 (21.2) |
| Poultry | 9.77 (10.0) | 10.9 (10.3) | 11.7 (11.3) | 10.8 (11.1) | 11.4 (10.5) | 11.2 (10.7) |
| Red meat | 36.6 (57.2) | 30.8 (40.7) | 28.0 (30.8) | 33.1 (46.4) | 28.4 (34.4) | 29.1 (34.4) |
| Processed meat | 61.9 (63.9) | 54.9 (56.8) | 51.6 (48.6) | 60.4 (64.7) | 51.8 (51.2) | 53.0 (51.0) |
| Offals | 0.92 (1.41) | 0.88 (1.37) | 0.87 (1.28) | 0.89 (1.39) | 0.87 (1.38) | 0.88 (1.33) |
| Condiments | 29.3 (17.1) | 28.7 (16.6) | 28.9 (15.2) | 28.5 (17.4) | 28.6 (15.2) | 29.1 (15.6) |
| Soups | 43.3 (35.4) | 44.4 (36.3) | 44.8 (36.9) | 42.9 (36.7) | 45.2 (37.1) | 44.6 (36.4) |
| Miscellaneous | 0.44 (0.23) | 0.45 (0.25) | 0.45 (0.24) | 0.44 (0.23) | 0.45 (0.24) | 0.45 (0.24) |

FUP3 - follow-up 3

**Supplementary Table 6 Association between the baseline HDS and incident type 2 diabetes, myocardial infarction and stroke within a mean follow-up of 11 years**

|  |  | HR for the comparison of quintiles of the baseline HDS | | | | |  |  |
| --- | --- | --- | --- | --- | --- | --- | --- | --- |
|  | **n/n_cases_** | **Q1** | **Q2** | **Q3** | **Q4** | **Q5** | **HR per 1 SD** | |
| **Type 2 diabetes** | 22,255/1,430 | Ref | 1.00 (0.85 – 1.16) | 1.01 (0.86 – 1.19) | 0.84 (0.71 – 1.00) | 0.82 (0.69 – 0.97) | 0.90 (0.85 – 0.96) | |
| **Myocardial infarction** | 23,111/331 | Ref | 1.03 (0.75 – 1.40) | 0.85 (0.61 – 1.20) | 0.90 (0.64 – 1.26) | 0.89 (0.62 – 1.27) | 0.98 (0.86 – 1.11) | |
| **Stroke** | 23,248/350 | Ref | 0.72 (0.53 – 0.99) | 0.91 (0.67 – 1.23) | 0.71 (0.51 – 0.99) | 0.57 (0.40 – 0.82) | 0.83 (0.73 – 0.94) | |

HDS – Healthy Diet Score; HR – Hazard ratio; SD – standard deviation; Cox Proportional Hazards Regression model was adjusted for sex, education, occupation, vitamin supplementation, self-reported hypertension, smoking status, physical activity, lifetime alcohol consumption, total energy intake and BMI, all measured at baseline.

**Supplementary Table 7 Stratified analyses by sex on the prospective associations between change in the diet quality scores and chronic disease risk**

| Outcome | Diet score |  | N | N_cases_ | HR per 20% increase | P for interaction |
| --- | --- | --- | --- | --- | --- | --- |
| Type 2 diabetes | **MedPyr** |  |  |  |  |  |
|  |  | Men | 7,931 | 285 | 1.00 (0.88 – 1.14) | 0.18 |
|  |  | Women | 13,462 | 283 | 0.89 (0.77 – 1.04) |  |
|  | **HDS** |  |  |  |  |  |
|  |  | Men | 7,931 | 285 | 1.00 (0.90 – 1.11) | 0.23 |
|  |  | Women | 13,462 | 283 | 0.91 (0.79 – 1.06) |  |
| Myocardial infarction | **MedPyr** |  |  |  |  |  |
|  |  | Men | 8,646 | 112 | 1.03 (0.84 – 1.28) | 0.65 |
|  |  | Women | 14,305 | 59 | 0.85 (0.58 – 1.16) |  |
|  | **HDS** |  |  |  |  |  |
|  |  | Men | 8,646 | 112 | 1.01 (0.84 – 1.21) | 0.58 |
|  |  | Women | 14,305 | 59 | 1.04 (0.77 – 1.41) |  |
| Stroke | **MedPyr** |  |  |  |  |  |
|  |  | Men | 8,867 | 110 | 0.85 (0.69 – 1.04) | 0.60 |
|  |  | Women | 14,220 | 79 | 0.99 (0.74 – 1.33) |  |
|  | **HDS** |  |  |  |  |  |
|  |  | Men | 8,867 | 110 | 0.91 (0.77 – 1.08) | 0.61 |
|  |  | Women | 14,220 | 79 | 0.92 (0.70 – 1.20) |  |

HDS – Healthy Diet Score; HR – Hazard ratio; MedPyr – Mediterranean Pyramid; Cox Proportional Hazards Regression model was adjusted for baseline diet score and baseline education, baseline and FUP3 information on: occupation, smoking status, self-reported hypertension, lifetime alcohol consumption, total energy intake, physical activity (updated information only available at FUP2), BMI

**Supplementary Table 8 Stratified analyses by baseline diet quality score tertiles on the prospective associations between the diet score changes and chronic disease risk**

| Outcome | Baseline diet quality score tertiles | N | N_cases_ | HR per 20% increase | P for interaction |
| --- | --- | --- | --- | --- | --- |
| Type 2 diabetes | **MedPyr** |  |  |  | 0.28 |
|  | Low (2.15 – 5.95) | 6,951 | 223 | 0.97 (0.87 – 1.08) |  |
|  | Medium (5.95 – 6.99) | 7,149 | 187 | 0.83 (0.69 – 1.01) |  |
|  | High (6.99 – 11.25) | 7,293 | 158 | 1.18 (0.92 – 1.51) |  |
|  | **HDS** |  |  |  | 0.65 |
|  | Low (1.25 – 4.00) | 7,223 | 218 | 0.98 (0.89 – 1.08) |  |
|  | Medium (4.08 – 4.75) | 7,241 | 184 | 1.09 (0.91– 1.31) |  |
|  | High (4.83 – 8.13) | 6,929 | 166 | 1.03 (0.81 – 1.30) |  |
| Myocardial infarction | **MedPyr** |  |  |  | 0.16 |
|  | Low (2.15 – 5.95) | 7,612 | 68 | 1.06 (0.87 – 1.28) |  |
|  | Medium (5.95 – 6.99) | 7,642 | 49 | 0.84 (0.58 – 1.22) |  |
|  | High (6.99 – 11.25) | 7,697 | 54 | 0.98 (0.65 – 1.49) |  |
|  | **HDS** |  |  |  | 0.13 |
|  | Low (1.25 – 4.00) | 7,785 | 69 | 1.07 (0.92 – 1.25) |  |
|  | Medium (4.08 – 4.75) | 7,777 | 55 | 0.66 (0.49 – 0.91) |  |
|  | High (4.83 – 8.13) | 7,389 | 47 | 1.16 (0.74 – 1.82) |  |
| Stroke | **MedPyr** |  |  |  | 0.76 |
|  | Low (2.15 – 5.95) | 7,660 | 77 | 0.90 (0.75 – 1.09) |  |
|  | Medium (5.95 – 6.99) | 7,690 | 55 | 0.78 (0.55 – 1.11) |  |
|  | High (6.99 – 11.25) | 7,737 | 57 | 1.01 (0.67 – 1.51) |  |
|  | **HDS** |  |  |  | 0.51 |
|  | Low (1.25 – 4.00) | 7,751 | 74 | 1.03 (0.88 – 1.21) |  |
|  | Medium (4.08 – 4.75) | 7,757 | 72 | 1.00 (0.75 – 1.35) |  |
|  | High (4.83 – 8.13) | 7,390 | 43 | 0.83 (0.52 – 1.32) |  |

HDS – Healthy Diet Score; HR – Hazard ratio; MedPyr – Mediterranean Pyramid; Cox Proportional Hazards Regression model was adjusted for sex, baseline education, baseline and FUP3 information on: occupation, smoking status, self-reported hypertension, lifetime alcohol consumption, total energy intake, physical activity (updated information only available at FUP2), BMI

**Supplementary Table 9 Sensitivity analyses on the prospective associations between the change in the two diet quality scores and the three disease outcomes**

| Exclusion of: | Outcome | N | Diet score |  | Decrease | Stable | Increase | Per 20% increase |
| --- | --- | --- | --- | --- | --- | --- | --- | --- |
| Implausible energy intake (n=675) | T2D | 20,777 | MedPyr | Cases/Person-years | 107/15,603 | 112/16,943 | 333/59,301 |  |
|  |  |  |  | Model 2 | 1.00 (0.76 – 1.31) | Ref | 0.74 (0.59 – 0.94) | 0.95 (0.86 – 1.04) |
|  |  |  | HDS | Cases/Person-years | 76/13,006 | 83/13,660 | 393/65,181 |  |
|  |  |  |  | Model 2 | 0.98 (0.71 – 1.34) | Ref | 0.89 (0.69 - 1.15) | 0.96 (0.87 – 1.05) |
|  | MI | 22,296 | MedPyr | Cases/Person-years | 31/17,013 | 30/18,241 | 105/63,993 |  |
|  |  |  |  | Model 2 | 0.93 (0.56 – 1.55) | Ref | 0.96 (0.62 – 1.48) | 0.98 (0.83 – 1.17) |
|  |  |  | HDS | Cases/Person-years | 28/13,849 | 23/14,794 | 115/70,604 |  |
|  |  |  |  | Model 2 | 1.19 (0.68 – 2.08) | Ref | 0.95 (0.58 – 1.53) | 1.02 (0.87 – 1.19) |
|  | Stroke | 22,422 | MedPyr | Cases/Person-years | 42/17,031 | 26/18,424 | 117/64,266 |  |
|  |  |  |  | Model 2 | 1.61 (0.98 – 2.64) | Ref | 1.21 (0.77 – 1.90) | 0.90 (0.75 – 1.06) |
|  |  |  | HDS | Cases/Person-years | 26/13,967 | 19/14,855 | 137/70,899 |  |
|  |  |  |  | Model 2 | 1.73 (0.97 – 3.09) | Ref | 1.14 (0.69 – 1.90) | 0.92 (0.80 – 1.07) |
| Diet change due to weight gain (n=5,536) | T2D | 15,559 | MedPyr | Cases/Person-years | 78/12,495 | 73/12,940 | 212/43,319 |  |
|  |  |  |  | Model 2 | 1.07 (0.78 – 1.49) | Ref | 0.75 (0.56 – 1.00) | 0.92 (0.81 – 1.04) |
|  |  |  | HDS | Cases/Person-years | 53/10,652 | 59/10,440 | 251/47,662 |  |
|  |  |  |  | Model 2 | 0.85 (0.58 – 1.23) | Ref | 0.81 (0.59 – 1.11) | 0.91 (0.82 – 1.02) |
|  | MI | 16,552 | MedPyr | Cases/Person-years | 29/13,491 | 25/13,733 | 85/46,335 |  |
|  |  |  |  | Model 2 | 1.00 (0.58 – 1.72) | Ref | 1.01 (0.63 – 1.62) | 0.99 (0.82 – 1.20) |
|  |  |  | HDS | Cases/Person-years | 23/11,209 | 21/11,199 | 95/51,151 |  |
|  |  |  |  | Model 2 | 1.01 (0.56 – 1.84) | Ref | 0.98 (0.59 – 1.63) | 1.03 (0.87 – 1.22) |
|  | Stroke | 16,619 | MedPyr | Cases/Person-years | 34/13,520 | 17/13,867 | 83/46,474 |  |
|  |  |  |  | Model 2 | 1.91 (1.06 – 3.43) | Ref | 1.31 (0.76 – 2.26) | 0.90 (0.74 – 1.09) |
|  |  |  | HDS | Cases/Person-years | 24/11,302 | 13/11,264 | 97/51,295 |  |
|  |  |  |  | Model 2 | 1.95 (0.99 – 3.84) | Ref | 1.35 (0.73 – 2.48) | 0.91 (0.77 – 1.08) |
| Diet change due to high blood pressure  (n = 143) | T2D | 19,333 | MedPyr | Cases/Person-years | 96/14,927 | 102/15,992 | 293/54,641 |  |
|  |  |  |  | Model 2 | 0.96 (0.72 – 1.27) | Ref | 0.77 (0.60 – 0.98) | 0.97 (0.87 – 1.08) |
|  |  |  | HDS | Cases/Person-years | 71/12,770 | 74/12,822 | 346/59,970 |  |
|  |  |  |  | Model 2 | 0.93 (0.67 – 1.29) | Ref | 0.87 (0.66 – 1.14) | 0.96 (0.87 – 1.06) |
|  | MI | 20,587 | MedPyr | Cases/Person-years | 31/16,130 | 27/17,061 | 99/58,545 |  |
|  |  |  |  | Model 2 | 1.03 (0.61 – 1.74) | Ref | 1.04 (0.67 – 1.64) | 0.99 (0.83 – 1.18) |
|  |  |  | HDS | Cases/Person-years | 25/13,483 | 22/13,781 | 110/64,473 |  |
|  |  |  |  | Model 2 | 1.04 (0.58 – 1.85) | Ref | 1.02 (0.62 – 1.67) | 1.04 (0.88 – 1.22) |
|  | Stroke | 20,691 | MedPyr | Cases/Person-years | 41/16,192 | 21/17,234 | 105/58,752 |  |
|  |  |  |  | Model 2 | 1.91 (1.12 – 3.24) | Ref | 1.43 (0.88 – 2.34) | 0.94 (0.79 – 1.13) |
|  |  |  | HDS | Cases/Person-years | 31/13,594 | 16/13,868 | 120/64,717 |  |
|  |  |  |  | Model 2 | 2.07 (1.13 – 3.79) | Ref | 1.23 (0.71 – 2.14) | 0.91 (0.78 – 1.07) |
| Diet change due to diabetes (n = 766) | T2D | 19,387 | MedPyr | Cases/Person-years | 94/14,954 | 102/16,045 | 291/54,814 |  |
|  |  |  |  | Model 2 | 0.94 (0.70 – 1.25) | Ref | 0.77 (0.60 – 0.98) | 0.97 (0.87 – 1.08) |
|  |  |  | HDS | Cases/Person-years | 71/12,802 | 74/12,846 | 342/60,165 |  |
|  |  |  |  | Model 2 | 0.92 (0.67 – 1.29) | Ref | 0.86 (0.65 – 1.13) | 0.96 (0.87 – 1.05) |
|  | MI | 20,226 | MedPyr | Cases/Person-years | 30/15,852 | 27/16,788 | 96/57,590 |  |
|  |  |  |  | Model 2 | 0.98 (0.58 – 1.67) | Ref | 1.00 (0.64 – 1.57) | 0.97 (0.81 – 1.16) |
|  |  |  | HDS | Cases/Person-years | 24/13,329 | 21/16,588 | 108/63,313 |  |
|  |  |  |  | Model 2 | 1.05 (0.58 – 1.90) | Ref | 1.01 (0.61 – 1.67) | 1.01 (0.85 – 1.18) |
|  | Stroke | 20,321 | MedPyr | Cases/Person-years | 39/15,898 | 19/16,962 | 99/57,792 |  |
|  |  |  |  | Model 2 | 2.01 (1.15 – 3.49) | Ref | 1.47 (0.88 – 2.45) | 0.95 (0.78 – 1.14) |
|  |  |  | HDS | Cases/Person-years | 30/13,432 | 14/13,675 | 113/63,546 |  |
|  |  |  |  | Model 2 | 2.28 (1.20 – 4.32) | Ref | 1.37 (0.76 – 2.46) | 0.91 (0.77 – 1.07) |
| Diet change due to dyslipidemia (n = 596) | T2D | 19,018 | MedPyr | Cases/Person-years | 94/14,724 | 101/15,762 | 288/53,643 |  |
|  |  |  |  | Model 2 | 0.95 (0.71 – 1.26) | Ref | 0.76 (0.60 - 0.98) | 0.97 (0.88 – 1.08) |
|  |  |  | HDS | Cases/Person-years | 70/12,622 | 72/12,623 | 341/58,885 |  |
|  |  |  |  | Model 2 | 0.94 (0.67 – 1.31) | Ref | 0.88 (0.67 – 1.17) | 0.97 (0.88 – 1.07) |
|  | MI | 20,280 | MedPyr | Cases/Person-years | 32/15,922 | 27/16,831 | 99/57,554 |  |
|  |  |  |  | Model 2 | 1.06 (0.63 – 1.78) | Ref | 1.05 (0.67 – 1.64) | 0.98 (0.82 – 1.17) |
|  |  |  | HDS | Cases/Person-years | 26/13,340 | 22/13,567 | 110/63,400 |  |
|  |  |  |  | Model 2 | 1.08 (0.61 – 1.92) | Ref | 1.01 (0.62 – 1.65) | 1.02 (0.87 – 1.20) |
|  | Stroke | 20,380 | MedPyr | Cases/Person-years | 40/15,977 | 21/16,990 | 103/57,770 |  |
|  |  |  |  | Model 2 | 1.85 (1.09 – 3.15) | Ref | 1.40 (0.85 – 2.29) | 0.94 (0.79 – 1.13) |
|  |  |  | HDS | Cases/Person-years | 30/13,446 | 16/13,654 | 118/63,638 |  |
|  |  |  |  | Model 2 | 1.98 (1.08 – 3.65) | Ref | 1.20 (0.69 – 2.09) | 0.92 (0.79 – 1.08) |
| Alcohol component from MedPyr | T2D | 21,393 | MedPyr | Cases/Person-years | 113/14,374 | 104/17,492 | 351/62,643 |  |
|  |  |  |  | Model 2 | 1.25 (0.95 – 1.63) | Ref | 0.83 (0.65 – 1.05) | 0.90 (0.81 – 1.00) |
|  | MI | 22,951 | MedPyr | Cases/Person-years | 29/15,664 | 29/18,814 | 113/67,624 |  |
|  |  |  |  | Model 2 | 0.98 (0.58 – 1.64) | Ref | 1.08 (0.69 – 1.67) | 0.99 (0.83 – 1.19) |
|  | Stroke | 23,087 | MedPyr | Cases/Person-years | 39/15,699 | 39/18,982 | 111/67,935 |  |
|  |  |  |  | Model 2 | 1.12 (0.72 – 1.76) | Ref | 0.68 (0.45 – 1.01) | 0.83 (0.70 – 1.00) |

HDS – Healthy Diet Score; MedPyr – Mediterranean Pyramid; MI – Myocardial infarction; T2D – Type 2 Diabetes;

**Supplementary Table 10 Overview of different sources to identify incident cases of T2D, MI and stroke in the EPIC-Potsdam study**

| Sources | T2D | MI | Stroke |
| --- | --- | --- | --- |
| Self-report | 89% | 84% | 79% |
| Medication | 4% | - | - |
| Diet change due to diabetes | 1% | - | - |
| Others* | 4% | 7% | 3% |
| Death certificate | 2% | 9% | 12% |
| Reported symptom | - | - | 3% |
| TIA self-report | - | - | 3% |

Displayed are the relative frequencies of sources according to each of the three disease outcomes. *Others includes tumor center, patient files, telephone interview, suspicion facts; MI – Myocardial infarction; T2D – Type 2 Diabetes; TIA – Transitory Ischaemic Attack

References

1. Nöthlings U (2004) Development and evaluation of an abbreviated food frequency questionnaire to discriminate between study participants in a cohort study. Dissertation, Technical University Berlin, Berlin

2. Slimani N, Valsta L, Group E (2002) Perspectives of using the EPIC-SOFT programme in the context of pan-European nutritional monitoring surveys: methodological and practical implications. Eur J Clin Nutr 56 Suppl 2:S63-74. doi:10.1038/sj.ejcn.1601430

3. Galbete C, Kroger J, Jannasch F, Iqbal K, Schwingshackl L, Schwedhelm C, Weikert C, Boeing H, Schulze MB (2018) Nordic diet, Mediterranean diet, and the risk of chronic diseases: the EPIC-Potsdam study. BMC Med 16 (1):99. doi:10.1186/s12916-018-1082-y

4. Jannasch F, Nickel DV, Bergmann MM, Schulze MB (2022) A New Evidence-Based Diet Score to Capture Associations of Food Consumption and Chronic Disease Risk. Nutrients 14 (11). doi:10.3390/nu14112359
